# The Criminal Law Amendment Bill

**Published:** 1917-03-03

**Authors:** 


### THE CRIMINAL LAW AMENDMENT BILL.

### State Recognition of Preventive Medicine.

"There is some soul of goodness in things evil." For all its horrors and the indescribable and immeasurable misery that it has already caused, the war has done some good, and in some respects the good it has done is very great. It has shaken us up and taught us all that we are living in a world of realities, and that to pretend, as we have been for many years pretending, that we are living in a world of fiction; to pretend that things that exist do not exist; to pretend that things of the utmost moment are of no importance at all; to pretend that things of despicable triviality are the only things that matter, is a disastrous folly that can only end in ruin. It is owing to the war that we have seen the portentous spectacle, which no man living ever expected to see, of officials appointed to posts on no other ground than that they were competent to fulfil the duties and knew something about them. This is in the political world more than the equivalent of an earthquake in the physical world. It leaves us wondering whether we are indeed in the same world that we have grown up in and known from our youth. Until this war the conditions of success in political life have been a glib tongue, a rhinoceros hide, an exalted self-sufficiency, and an absence of shame; and we no more expected to see this altered than we expected to see the sun on some portentous morning rise out of the western horizon.

### Realities *v.* Shams.

However, the impossible has happened; and not in that respect only. We may now actually recognise the existence of other realities which we have always pretended were non-existent. In time of peace there is no more dreadful scourge of the human race than venereal diseases. They are met with at every turn; they stand third on the list of causes of death; and they do not cause death with merciful rapidity. Death is by no means the greatest of the evils that they produce. They menace not only the health, the lives, and the happiness of individuals, but also the vigour of the race, and even its continuation. They are responsible for miscarriage, abortion, and sterility; for the entry into the world of children predestined to be a misery to themselves and a burden to others; for children born with a heritage of blindness, deafness, repulsive ugliness, idiocy, imbecility, feeble-mindedness, or insanity. But in the world of shams in which we lived before the war all this was ignored. It was put aside as improper. It was not to be spoken of. We had to pretend that it did not exist; just as we had to pretend that a cosmopolitan lawyer was the proper person to be Minister of War, and a dilettante writer of third-rate essays a proper person to govern a disaffected province. The war has taught us at long length that after all this world is a world of realities, and that to pretend that it is a world of shams is to invite disaster. Disaster is sometimes slow to respond to the invitation, but it never fails if time is given, and so Mr. Birrell found. In the matter of venereal disease, disaster was quick to respond. It has been invited, and how it has responded to the invitation tens of thousands of homes testify—homes in which a would-be mother is barren, or if she has borne children they are a horror to her, and she prays that she may bear no more. Homes in which the father is unable even to do a fair day's work, and at intervals more or less frequent is unable to work at all, brings up his children therefore in poverty, ill-clothed and ill-fed, and dies early to leave them a burden on others.

At last, however, this sham, which was always known to be a sham, is exploded, and we are free to express our belief in its reality, and even to take measures against it. The war has forced us to recognise that venereal disease exists, and has forced us partly by its general influence in abolishing shams and compelling us to see things as they are, and partly by bringing the matter so prominently forward and thrusting it under our noses so that it was impossible to pretend any longer that we did not see it. The *Morning Post*, all honour to it, took the matter up and thrust it before the eyes of the public. Other newspapers followed suit, and a Royal Commission was appointed to investigate and report. So destructive of shams is the influence of the war that even the Royal Commission was not a sham. It did its work thoroughly, and it did it promptly. It made valuable suggestions for coping with the evil, and these await action.

### Prevention Better than Cure.

But the treatment of disease after it has been contracted is after all only a palliative. The real remedy for this, as for every other disease, is prevention. These diseases ought to be prevented, can be prevented, and must be prevented. Physically, their prevention is a much easier matter than the prevention of tuberculosis, and we have already gone some way towards the prevention of tuberculosis. Tuberculosis is contracted in many ways, not only through the food, from which it is difficult to prevent the bacillus from obtaining access, but also through the air, which cannot in large centres of population be kept entirely free. The venereal diseases are communicated only by direct contagion, and if contact is prevented are incommunicable. Moreover, even if contact does take place, infection may be prevented. The Criminal Law Amendment Bill is intended to secure several very different objects. If not an omnibus Bill it is a plurimis Bill; but its main purpose is to prevent the communication of venereal diseases, and to prevent them by preventing that mode of contact which is the chief means of communication. That its provisions will prevent altogether the spread of these diseases is chimerical, and is not expected even by their authors; but it is intended and expected to diminish the spread by diminishing the opportunities for it. How far its provisions are likely to attain this end we shall examine in a subsequent issue.

